# Breastfeed4Ghana: Design and evaluation of an innovative social media campaign

**DOI:** 10.1111/mcn.12909

**Published:** 2019-12-22

**Authors:** Kassandra Harding, Richmond Aryeetey, Grace Carroll, Opeyemi Lasisi, Rafael Pérez‐Escamilla, Marissa Young

**Affiliations:** ^1^ Yale School of Public Health New Haven Connecticut; ^2^ School of Public Health University of Ghana Accra Ghana; ^3^ Columbia University New York New York

**Keywords:** breastfeeding, campaign, feasibility, Ghana, promotion, social media

## Abstract

Although targeting health behaviour change through social media campaigns has gained traction in recent years, few studies have focused on breastfeeding social media campaigns. Within the context of rising social media utilization and recent declines in exclusive breastfeeding practices in Ghana, we implemented *Breastfeed4Ghana*, a Facebook‐ and Twitter‐based breastfeeding social media campaign. This study determined feasibility of implementing Breastfeed4Ghana and evaluated its impact on breastfeeding knowledge in Ghana. Key performance indicators of the campaign were monitored on social media platforms, Facebook and Twitter. An online cross‐sectional survey conducted across three time points (*n* = 451) assessed breastfeeding knowledge, campaign exposure, and understanding and acceptability of Breastfeed4Ghana among Ghanaian adults. Modified Poisson models were used to assess the relationship between campaign exposure and breastfeeding knowledge, adjusting for survey time point, sex, and parenthood status. The campaign acquired 4,832 followers. Based on follower demographics collected from Facebook and Twitter analytics, the target population was successfully reached. Campaign exposure among survey participants was 42.3% and 48.7% at midline and endline, respectively. Campaign acceptability was high (>90%), and >44% of those exposed to the campaign also shared the campaign with others. However, 61.0% of those exposed did not know or could not remember the purpose of the campaign. Campaign exposure was not associated with higher breastfeeding knowledge (APR [95% confidence interval] = 0.96 [0.73, 1.26]). Breastfeed4Ghana was highly feasible. However, campaign understanding yielded mixed findings and may explain the limited impact on breastfeeding knowledge.

**Key Messages**

- Social media‐based health behaviour campaigns are feasible and acceptable in sub‐Saharan Africa and can serve as a reliable source of health information on the internet.
- Campaign acceptability was high; however, campaign exposure was not associated with improved breastfeeding knowledge.
- Campaign understanding, exposure, and engagement may be increased with a higher frequency of campaign materials being posted and more precise targeting.

## 1 INTRODUCTION

The health benefits of breastfeeding to both the child and mother are well established (Victora et al., 2016); yet in Ghana, the prevalence of exclusive breastfeeding among infants under 6 months old declined from 63% in 2008 to 52% in 2014 (Ghana Statistical Service, Ghana Health Service and I. International, 2015). In response, a committee of Ghanaian breastfeeding experts and stakeholders led by the University of Ghana implemented the Becoming Breastfeeding Friendly (BBF) Initiative. The BBF Initiative provides countries with a toolbox for assessing their breastfeeding scale‐up environment based on the breastfeeding gear model and developing recommendations to strengthen their breastfeeding environment (Perez‐Escamilla, Curry, Minhas, Taylor, & Bradley, 2012; Aryeetey et al., 2018). Key recommendations from the BBF process included strengthening advocacy, harnessing support for maternity protection laws, and providing more effective dissemination of accurate actionable information about breastfeeding (Aryeetey et al., 2018). Additionally, the BBF committee emphasized utilizing cost‐effective approaches to act on such recommendations, including through social media (Carroll et al., 2019).

Health behaviour change interventions facilitated through social media are on the rise, with a concentration of research within the context of high‐income countries (Webb, Joseph, Yardley, & Michie, 2010, Laranjo et al., 2015, Maher, Ryan, Kernot, Podsiadly, & Keenihan, 2016, Yang, 2017). Social media has penetrated communities across the globe, and usage has recently increased in developing economies (Pew Research Center, 2018). Although the typical social media user is younger, more educated, and wealthier, trends across Africa indicate rapid adoption is shifting towards the middle class (Pew Research Center, 2016).

Between 2015 and 2017, internet use among adults in Ghana increased from 25% to 39%, and social media penetration increased by 60%, from 20% to 32% (Pew Research Center, 2016; Pew Research Center, 2018). Some sectors in Ghana have recognized the potential of social media as a tool to reach a wide audience for change (Pen Plus Bytes, 2016). However, the health sector, including specifically breastfeeding, has not effectively generated a social media presence.

In the Web 2.0 era, social media has become a prominent source of health information for the public (Moorhead et al., 2013; Giustini, 2006). Breastfeeding support is no exception. In a qualitative study among New Zealand mothers, most participants reported seeking breastfeeding advice online and “weak ties” on Facebook (i.e., low proportion of overlapping friend networks) were notable information sources (Alianmoghaddam, Phibbs, & Benn, 2019). Instagram also represents a platform for a supportive breastfeeding environment, gleaned from an analysis of 4,089 Instagram images and 8,331 comments on breastfeeding, which were primarily positive (Marcon, Bieber, & Azad, 2019).

Specifically in Ghana, 68% of breastfeeding mothers surveyed in Accra planned to use social media for breastfeeding information (Okasha, 2018). Furthermore, researchers deployed an online survey among breastfeeding mothers in the United States and found that many breastfeeding mothers are on social media *while* breastfeeding (Tomfohrde & Reinke, 2016). Thus, social media has been recognized at the global level as a potentially effective platform for breastfeeding promotion (Perez‐Escamilla, 2012; Wolynn, 2012). Thus, in the context of Ghana, where social media utilization is growing exponentially and exclusive breastfeeding rates have decreased, social media has potential to reach a wide audience through a campaign that targets the protection, promotion, and support of breastfeeding.

Social media‐based campaigns are appealing because they offer a potentially more cost‐effective option than traditional marketing campaigns, especially when combined with strategies to acquire high exposure and engagement. Such campaigns also have the ability to reach a wide target audience and include fathers and families, which have been identified by mothers as important sources for breastfeeding information (Brown, 2016). However, a breastfeeding social media marketing campaign has not been implemented in the context of West Africa before, and the feasibility of such an intervention in this context is unknown. Similarly, there is a lack of information regarding best practices for a campaign of this nature, including expected impacts and indicators of success, although an extensive list of key performance indicators has been published (Neiger, Thackeray et al. 2012).

Based on the BBF committee findings and recommendations (Aryeetey et al., 2018), a social media‐targeted campaign on breastfeeding in Ghana was developed and tested through an iterative process between November 2017 and January 2018. For this campaign, three specific themes were designed to address key BBF recommendations, which highlighted protection, promotion, and support of breastfeeding. The objectives of the current study were to (a) determine feasibility of implementing a breastfeeding social media campaign in Ghana based on campaign reach, acceptability, and understanding and (b) evaluate the impact of exposure to the campaign on self‐reported breastfeeding knowledge.

## 2 METHODS

### 2.1 Description of campaign

**Figure 1:**
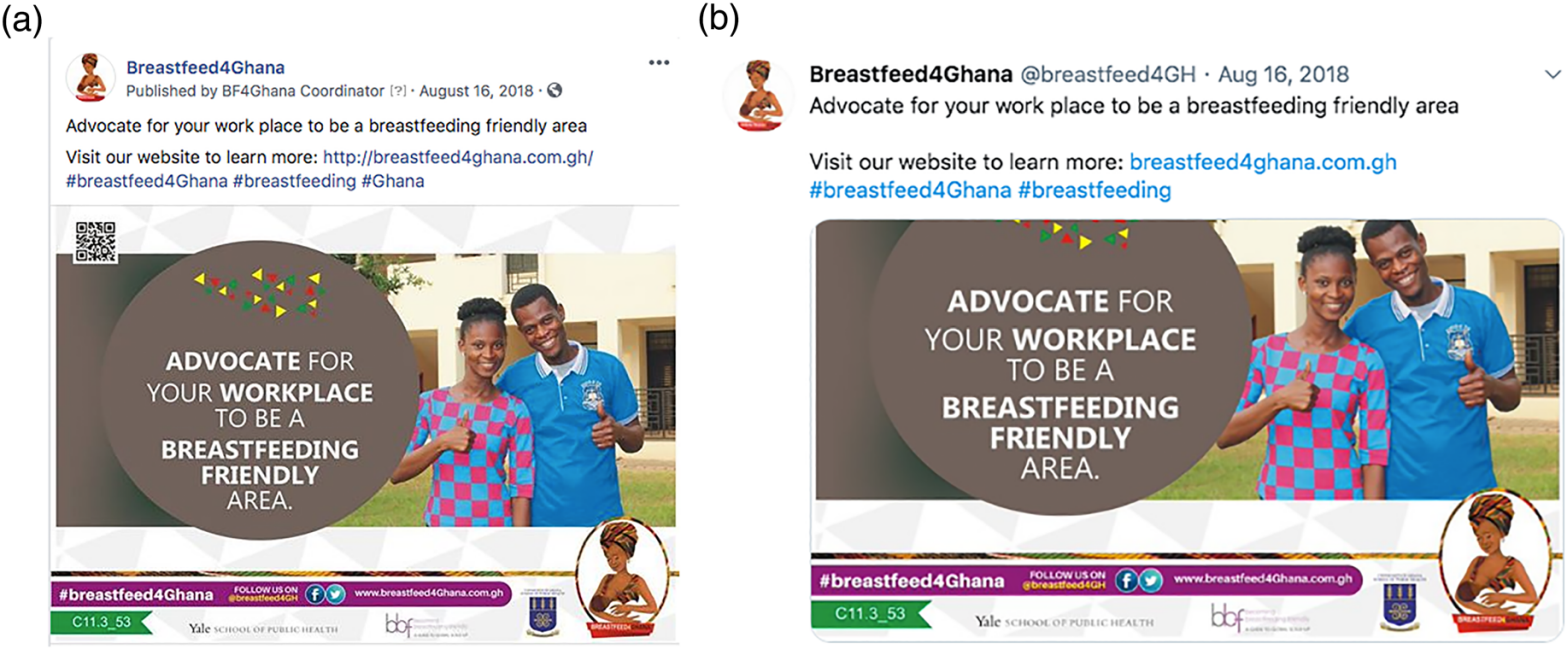
Example of a core campaign material post on Facebook (a) and Twitter (b)

Between March and September 2018, *Breastfeed4Ghana* was implemented as a 24‐week‐long breastfeeding social media campaign that targeted the protection, promotion, and support of breastfeeding in Ghana as derived from evidence‐informed BBF recommendations (Aryeetey et al., 2018). There were 60 core campaign materials, each consisting of an image with a concise and pertinent educational message (see Appendix A). Figure 1 depicts an example of a core campaign material posted on both Breastfeed4Ghana's Facebook and Twitter accounts. These core campaign materials were designed around three campaign themes: (a) *promote* correct information about breastfeeding; (b) *support* women to breastfeed anytime, anywhere; and (c) *protect* working women's right to breastfeed. This campaign was disseminated on social media platforms Facebook and Twitter using the Facebook page Breastfeed4Ghana and the Twitter handle @breastfeed4GH. Data from social@Ogilvy revealed that these two social media platforms were popular among social media users in Ghana at the time of the study (Social@Ogilvy, 2016), and the Pew Research Center revealed a growing penetration of social media in Ghana that reached 32% of the adult population in 2018 (Pew Research Center, 2018). Business accounts for both Facebook and Twitter were used to manage the dissemination of campaign materials (i.e., schedule automatic posts to a specified day and time) and access data analytics not available on standard user accounts. Additionally, a website was designed and maintained as the central platform for the campaign. This website housed information about the campaign, the 60 core campaign materials, resources organized by topics relevant to the campaign, and a weekly blog that provided more detailed information on topics related to the core campaign messages (http://breastfeed4ghana.com.gh).

The acquisition period occurred during the first 2 weeks of the campaign and aimed to create awareness of the campaign and acquire a base of social media followers for the campaign. During this period, promotional content about the campaign was shared at a high volume on Facebook and Twitter, averaging two posts per day on Facebook and three posts per day on Twitter. To create awareness about the campaign among the target population (i.e., the general population in Ghana), paid advertisements were posted through Facebook. Advertising through Twitter was unavailable during the acquisition period. The campaign was also promoted through social networks on WhatsApp (a freeware messaging app) among breastfeeding stakeholders and advocates, an advertisement on the University of Ghana website, and participation in two local radio interviews and a seminar at University of Ghana during the acquisition period and first month of the campaign.

On April 9, 2018, the campaign launched with the dissemination of the first core campaign material and an in‐person event with approximately 100 participants, including five high‐level government and non‐government stakeholders: the deputy director general of the Ghana Health Service, a nutrition specialist from UNICEF Ghana, the Greater Accra Regional Nutrition Officer, the Ghana Health Service Director of Health Promotion, and the Ghana Health Service Director of Child Health. This event was streamed on Facebook Live for the broader audience of Breastfeed4Ghana followers. During the following 12 weeks (Weeks 3 through 14 of the campaign), five core campaign materials were posted per week (Monday–Friday) on both Facebook and Twitter. Additionally, one blog was posted on the website and promoted on both social media platforms each week on Monday, and one video summarizing the campaign's activity for that week was shared on both platforms on Sunday.

Between Weeks 15 and 22 of the campaign, the top performing 40 core campaign materials were redisseminated via one of four different dissemination paths: (a) posted as usual (i.e., shared directly on campaign page), (b) reposted by key influencers who were identified by the size of their social media network, (c) reposted by randomly selected influencers, or (d) posted with paid advertisements. During these 8 weeks, five core campaign materials were posted per week (Monday–Friday), and additional posts included the weekly blog and video described above, a weekly quote from a key Ghanaian breastfeeding stakeholder (e.g., the First Lady, working mothers, and health professionals), and a weekly post highlighting a resource from the website.

The final 2 weeks of the campaign period (Weeks 23–24) focused on a video series called “Ask Ghana,” which included interviews filmed in Ghana with mothers, health professionals, and other local stakeholders. These videos aimed to increase engagement with the campaign and utilization of the website resources. The videos were posted three times a week and were supported through weekly complementary image–quote posts, blog posts, and resource posts that were typically posted daily.

### 2.2 Data: Campaign key performance indicators

Key performance indicator data for the campaign were collected based on each platform (i.e., Facebook page, Twitter page, and Website) and based on individual materials (i.e., Facebook posts, Tweets, and blog posts). Data were extracted from Facebook Insights, Twitter Analytics, and Google Analytics and entered into a Microsoft Access database. The analyses of key performance indicators presented in this article focus on the data extracted from Facebook and Twitter.

To assess the page performance and users following the campaign on Facebook and Twitter, the number of followers were collected weekly. On Facebook, this also included the number of page likes, total reach, total engagements, total page views, and demographic information on followers and users who engaged with the content posted (i.e., age category, country, and city). Similar performance data were not available on Twitter. Exposure, reach, and engagement are three key performance indicators commonly used in understanding social media in the context of health promotion (Neiger et al., 2012). Reach is most relevant in understanding page performance and can be measured by the number of page followers or page likes, as well as the demographics of the followers (i.e., age, sex, country, and city of residency). In this study, given the limitations of available data on Twitter, the primary metric for campaign page reach was weekly number of page followers.

For the data collection of core campaign materials (i.e., individual posts and tweets), key performance indicators were collected 1 day, 1 week, and 2 weeks after the material was posted and focused on exposure and engagement. Exposure was defined as Facebook *reach* (i.e., number of unique people who saw the content) and Twitter *impressions* (i.e., number of times a tweet appeared on a timeline and could be seen by a user more than once). Engagement included three subindicators calculated for each of Facebook and Twitter: applause rate (i.e., number of likes per number of followers), amplification rate (i.e., number of shares or retweets per number of followers), and conversation rate (i.e., number of comments or replies per number of followers). Therefore, Twitter impressions, Facebook reach, likes, shares, retweets, comments, and replies were collected for each post and merged with the corresponding weekly page data that included page followers. These data were collected at the same time points (i.e., 1 day, 1 week, and 2 weeks) for additional posts, such as blog, video, quote, and resource posts.

### 2.3 Data: Online survey

An online cross‐sectional survey was conducted at three time points: (a) baseline: 2 weeks prior to the initiation of the acquisition period, (b) midpoint: 13 weeks after the campaign launch, and (c) endline: at the end of the 24‐week campaign. The aim of this survey was to assess Facebook and Twitter users' exposure to the campaign, awareness and perception of the campaign, and breastfeeding knowledge. A convenient sample of Ghanaian adults were recruited via Facebook and Twitter advertisements that were standalone from the campaign social media pages and did not specifically target campaign followers.

Given the novelty of the study in Ghana and lack of relevant literature to inform a sample size calculation for the survey, a target sample size of 300 women and 150 men across the three time points was established. This sample size was based on feasibility for online recruitment of this population and an interest to oversample women as we anticipated that the campaign would be more appealing to women. Individuals were eligible to participate if they were ≥18 years of age, residing in Ghana, and had not taken the survey in the past month. The survey was self‐administered via Qualtrics®. Self‐reported breastfeeding knowledge was assessed from three multiple‐choice questions and four true/false questions that focused on key elements of information shared throughout the campaign. Campaign exposure was assessed based on participant report of viewing any material online regarding Breastfeed4Ghana. If participants reported exposure to the campaign, additional questions regarding the perceived purpose and characteristics of the campaign and the acceptability of the frequency of campaign posts were asked. Demographic characteristics and social media usage of participants was also collected.

All data were imported into Stata 13.1 (StataCorp, College Station, TX, USA) for cleaning and analysis.

### 2.4 Analytical approach

Feasibility of campaign implementation was explored through descriptive analyses of (a) the platform performance based on reach and cumulative reach over time, measured from monitoring data, and (b) understanding of the campaign purpose and acceptability of the campaign based on the cross‐sectional survey data.

To evaluate the impact of campaign exposure on breastfeeding knowledge, cross‐sectional survey data were pooled across the three time points. Breastfeeding knowledge was calculated as a score of correct responses to the seven knowledge‐based questions and dichotomized into “high knowledge” (≥6 questions correct) or “low knowledge” (<6 questions correct). Modified Poisson models were used to assess the relationship between campaign exposure and breastfeeding knowledge, adjusting for survey time point (i.e., baseline, midpoint, or endline), sex, and whether the participant was a parent of at least one child.

### 2.5 Ethical approval

This study was approved by the Yale University Institutional Review Board and Institutional Review Board at the Noguchi Memorial Institute for Medical Research at the University of Ghana (049/17‐18). Survey participants provided their electronic consent to participate in the survey prior to participation

## 3 RESULTS

### 3.1 Campaign feasibility and performance

**Figure 2:**
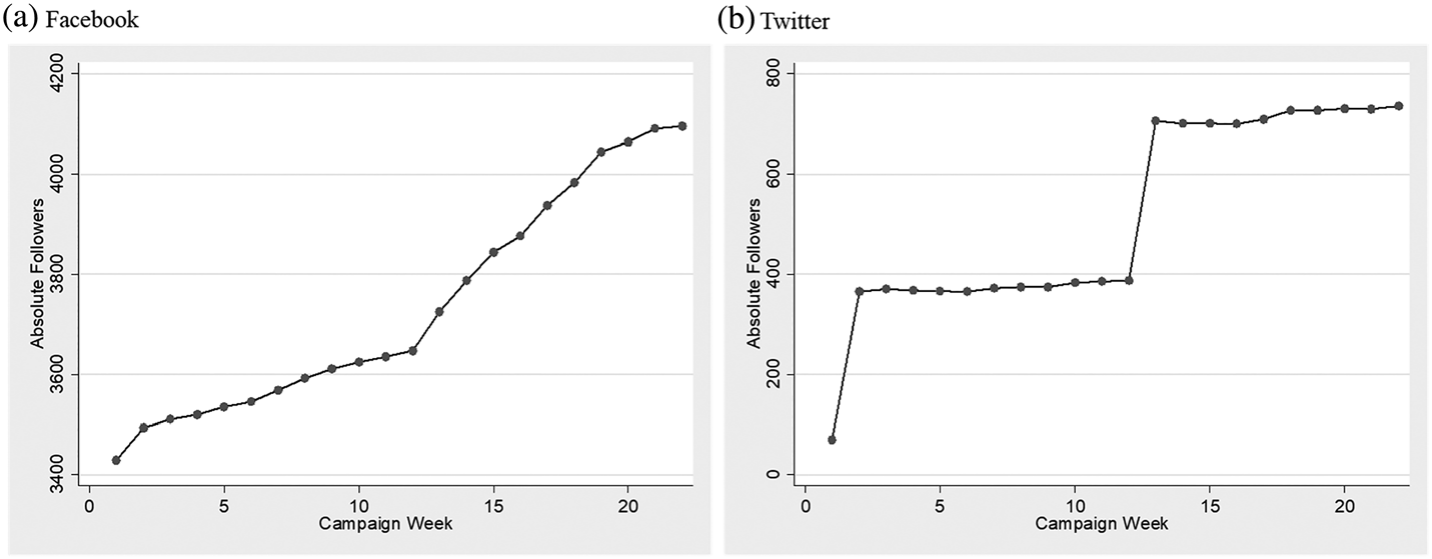
Absolute number of campaign followers on Facebook (a) and Twitter (b) across 22‐week active campaign period

During the acquisition period, a total of 3,061 followers were acquired on Facebook and 27 on Twitter, for a total of 3,088 followers at the campaign commencement. The small number of followers on Twitter was due to a delay in initiating paid advertising for the campaign on Twitter. The Facebook followers grew steadily over the initial 12 weeks and grew at a higher rate thereafter to 4,096 by the last week of the campaign. There were two spikes in Twitter follower growth due to targeted advertisement efforts, and the number of Twitter followers in the last week of the campaign was 736 (Figure 2). Therefore, the campaign reach grew from 3,061 to 4,832 followers by the end of the campaign period. Accounting for weekly follower growth over time, the cumulative campaign reach totalled 92,502 follower‐weeks across the 22‐week campaign period.

Demographics of Facebook followers shifted significantly between the start (Week 2) and end of the campaign (Week 24), with a higher prevalence of followers residing in Ghana (Week 2 vs. Week 24: 73.6% vs. 93.2%) and female (39% vs. 44%) by the end of the campaign. At the end of the campaign, 91.9% of followers were between 18 and 54 years of age, which was similar to that at the start of the campaign (92.2%). Demographic data of Twitter followers were not available.

### 3.2 Campaign exposure, understanding, acceptability, and engagement

**Figure 3:**
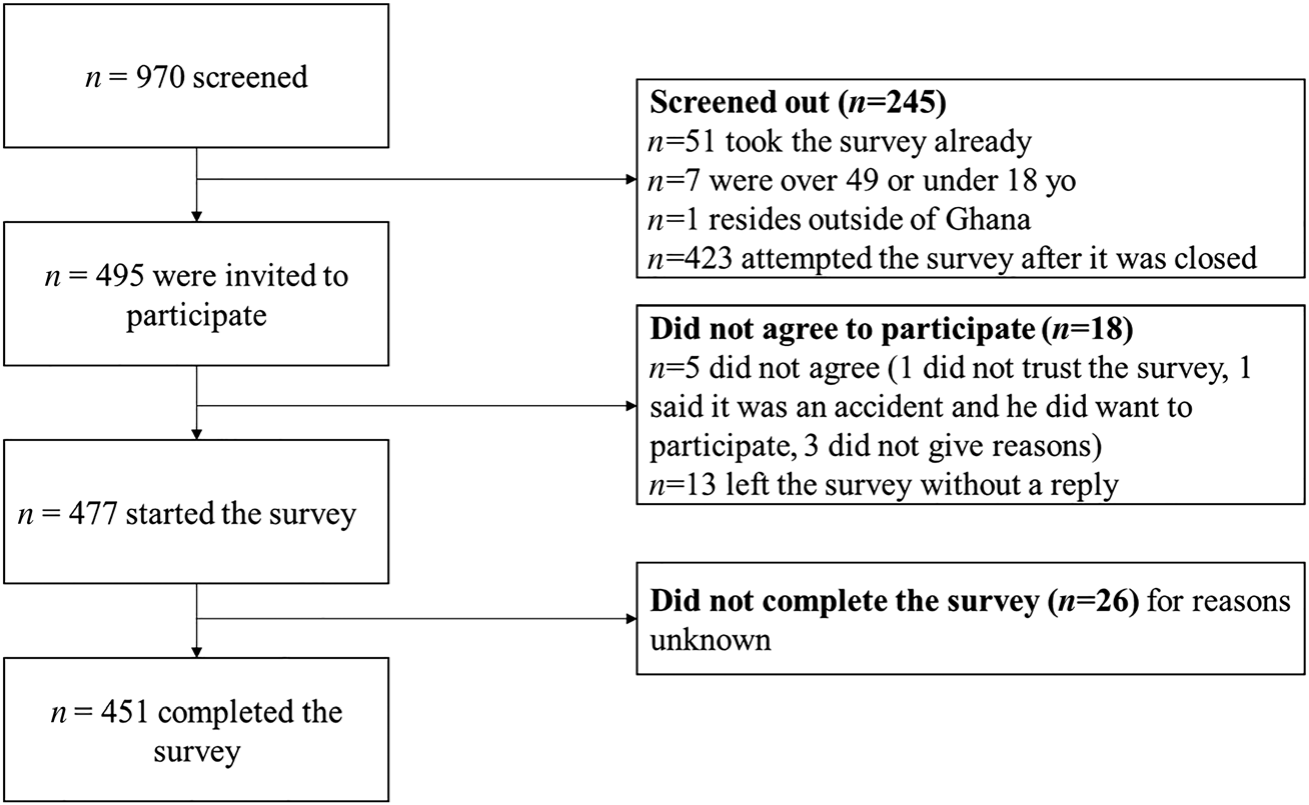
Flow diagram of survey participants

A total of 970 individuals were screened and 451 completed the survey at one of the three different time points the survey was administered (Figure 3). The participants were an average of 25.18 (*SD*: 4.55) years old, and the majority had never married (67.9%), had a Bachelor's degree or higher (77.2%), did not have children (78.3%), and had accessed the internet (90.2%), Facebook (58.9%), or Twitter (50.1%) daily (Table 1). Three months after the campaign launch, 42.3% (95% confidence interval, CI, [34.2, 50.6]) of participants reported exposure to the campaign and 48.7% [40.4, 57.0] reported exposure after 6 months. Among all the participants who took the survey, reported online exposure to topics related to Breastfeed4Ghana ranged from 30.8% [26.6, 35.3] exposed to breastfeeding resources to 76.7% [72.5, 80.5] exposed to child health topics (Table 2).

**Table 1:** Survey participant characteristics, aggregated (*n* = 451) and stratified by survey time point and participant sex

Characteristic	Total	Baseline	Midpoint	Endline	Male	Female
	*n* = 451	*n* = 152	*n* = 149	*n* = 150	*n* = 171	*n* = 280
Age (year)^a^	25.18 (4.55)	25.64 (4.89)	24.97 (4.42)	24.92 (4.30)	25.49 (4.54)	24.99 (4.55)
Region of Ghana						
Greater Accra	65.6	55.9	71.1	70	62.6	67.5
Western	2.9	2.0	4.0	2.7	2.9	2.9
Central	6.2	11.2	2.7	4.7	6.4	6.1
Volta	3.1	4.6	4	0.7	2.3	3.6
Eastern	3.6	2.6	5.4	2.7	2.3	4.3
Ashanti	10.2	13.1	8.1	9.3	14.0	7.9
Brong Ahafo	2.4	2.0	2.0	3.3	3.5	1.8
Northern	3.3	4.6	1.3	4.0	4.1	2.9
Upper East	2.0	2.6	1.3	2.0	1.2	2.5
Upper West	0.7	1.3	0	0.7	0.6	0.7
Never married	67.9	57.5	75.1	71.3	8.8	16.8
Employed^b^	40.1	42.8	40.9	36.7	38.0	41.4
Education: Bachelors or higher	77.2	70.4	81.2	80.0	80.1	75.4
Had children^c^^,^ d	21.7	27.6	15.4	22.0	11.1	28.2
Daily access in the past week to						
Internet, data, WiFi	90.2	89.5	89.9	91.3	91.2	89.6
Facebook	58.9	79.0	50.3	47.3	55.6	61.1
Twitter	50.1	19.7	65.1	66.0	66.1	40.4
Access to own smartphone	97.3	96.1	98.7	97.3	94.2	99.3

a Mean (*SD*).

b Of employed (*n* = 181): 96 were professional, 24 clerical, 28 sales, five skilled manual, three unskilled manual, one agriculture, and 24 other.

c Average number of children among those with a child (*n* = 98): 1.5(0.8); ranging from 1 to 5.

d Among those with at least one child (*n* = 98), all but three said at least one of their children breastfed.

**Table 2:** Exposure to materials online‐related key topics among online survey participants (% [95% CI])

Topic	Baseline (*n* = 152)	Midpoint (*n* = 149)	Endline (*n* = 150)	Total (*n* = 451)
Breastfeed4Ghana	—	42.3 [34.2, 50.6]	48.7 [40.4, 57.0]	30.2 [26.0, 34.6]
Breastfeeding resources	30.9 [23.7, 38.9]	31.5 [24.2, 39.7]	30.0 [22.8, 38.0]	30.8 [26.6, 35.3]
Baby formula	48.7 [40.5, 56.9]	43.0 [34.9, 51.3]	46.0 [37.8, 54.3]	45.9 [41.2, 50.6]
Baby food	71.1 [63.2, 78.1]	63.8 [55.5, 71.5]	64.7 [56.5, 72.3]	66.5 [62.0, 70.9]
Breast milk	61.8 [53.6, 69.6]	68.5 [60.3, 75.8]	68.7 [60.3, 75.8]	66.3 [60.6, 76.0]
Child health	77.6 [70.2, 84.0]	69.8 [61.7, 77.0]	82.7 [75.6, 88.4]	76.7 [72.5, 80.5]
Child feeding	72.4 [64.5, 79.3]	61.1 [52.8, 68.9]	71.3 [63.4, 78.4]	68.3 [63.8, 72.6]

Among those who reported exposure to the campaign, participants were asked what the campaign was about, with the option to check multiple responses. The majority understood that the campaign was about breastfeeding (88.6% [82.5, 94.7]), whereas 61.0% [51.7, 70.3] reported that they did not know or could not remember what the campaign was about, and 9.5% [3.9, 15.1] reported that they thought the campaign was related to infant formula (Table 3). At the same time, over 90% of those who reported exposure to the campaign reported that the campaign was informative, practical or useful, educational, or interesting, and 65.4% [56.8, 73.4] reported that the campaign was all of these descriptors (Table 3). Less than 5% reported that the campaign was confusing or dishonest, and 10.5% [5.3, 18.0] reported that the campaign was boring or unengaging. Approximately 44.8% [35.0, 54.8] of those who reported exposure to the campaign reported that they had shared the campaign material, with the majority having shared the material through WhatsApp (*n* = 19 out of 47) or by word of mouth (*n* = 16 out of 47; Table 3). When asked if the frequency of the campaign material seen was too much, not enough, or just right, the majority of participants reported that it was “not enough” (57.1% [47.1, 66.8]), and no participants reported that it was “too much.”

**Table 3:** Campaign understanding, acceptability, and engagement (among those exposed to the campaign at midpoint and endline *n* = 105^a^)

Campaign Indicators	% (95% CI)
Understanding	
What the campaign is about^b^	
Breastfeeding	88.6 [82.5, 94.7]
Don't remember	52.4 [42.8, 62.0]
Don't know	43.8 [34.3, 53.3]
Baby food	18.1 [10.7, 25.5]
Infant formula	9.5 [3.9, 15.1]
Acceptability	
Campaign described as	
Dishonest or untruthful	2.9 [0.6, 8.1]
Confusing	4.8 [1.6, 10.8]
Boring or unengaging	10.5 [5.3, 18.0]
Interesting	91.4 [84.4, 96.0]
Practical or useful	93.3 [86.7, 97.3]
Informative	97.1 [91.9, 99.4]
Education	99.1 [94.8, 100.0]
Frequency of campaign material described as	
Not enough	57.1 [47.1, 66.8]
Just right	42.9 [33.2, 52.9]
Engagement	
Shared campaign material^c^	44.8 [35.0, 54.8]
Visited the campaign website	29.5 [21.0, 39.2]

a Thirty‐one participants exposed to the campaign were not asked about the campaign (from the midpoint survey).

b Participants were asked what they thought the campaign was about and could respond with multiple answer. Only responses provided by >5% of participants is included in the table.

Among those who shared material (*n* = 47), 40% (19) shared through WhatsApp, 34% (16) by word of mouth, 30% (14) on Facebook, 21% (10) on Twitter, 4% (2) by email, and 2% (1) don't remember. Zero respondents said there was too much campaign information.

### 3.3 Impact of campaign exposure on breastfeeding knowledge

**Figure 4:**
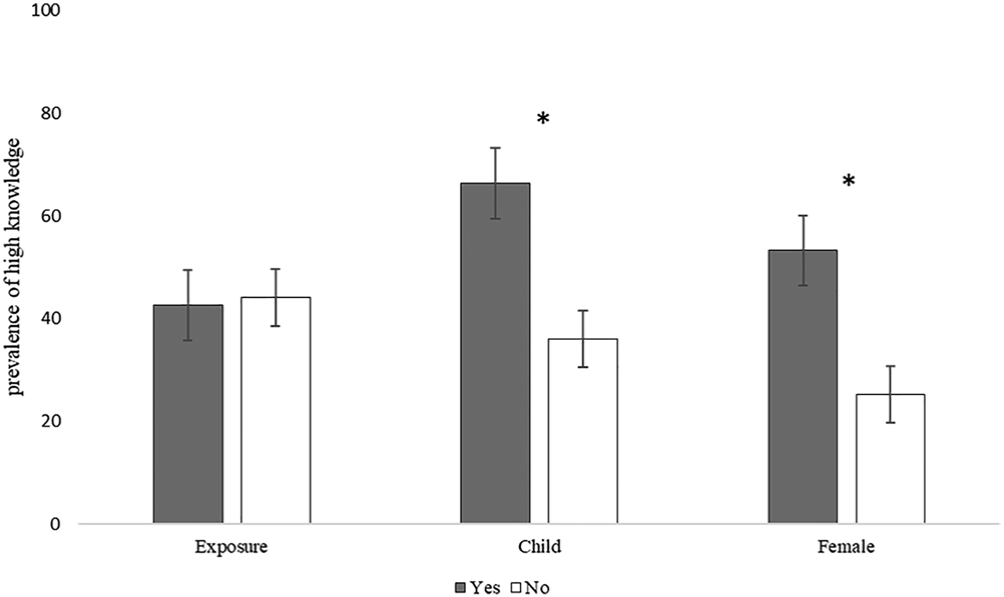
Prevalence of *high breastfeeding knowledge* ^†^ by campaign exposure and key participant characteristics represented with 95% confidence intervals. ^†^ = Correctly answered ≥6 out of seven breastfeeding knowledge questions. ^*^ = Indicates a significant difference (*P* < .05) in the crude prevalence of high breastfeeding knowledge based on bivariate analysis in a modified Poisson model

Of the seven survey questions that assessed breastfeeding knowledge, participants answered an average of 5.9 (*SD*: 1.2) questions correctly, and 42.6% [38.0, 47.3] answered six or seven correctly, indicating “high breastfeeding knowledge” (*n* = 451; Table 1). Approximately 39.0% [30.7, 47.7] of those who reported being exposed to the campaign (*n* = 136) had high breastfeeding knowledge, compared with 44.1% [38.5, 49.8] among those not exposed to the campaign (*n* = 315), with no significant differences by survey time point. In both unadjusted and adjusted models, high breastfeeding knowledge did not differ by reported campaign exposure (Figure 4; APR [95% CI] = 0.96 [0.73, 1.26]). However, both having a child (APR [95% CI]: 1.58 [1.28, 1.94]) and being female (1.93 [1.46, 2.55]) were associated with higher breastfeeding knowledge, compared with not having a child and being male, respectively.

In a post hoc power calculation using the given exposure to the campaign of 0.32, we had sufficient power to detect ~19% deviation in breastfeeding knowledge scores between those exposed to the campaign and those not exposed; thus, the study may have been underpowered.

## 4 DISCUSSION

This study successfully demonstrated the feasibility of implementing a breastfeeding social media marketing campaign in Ghana. The number of campaign followers grew throughout the active campaign period, with a cumulative reach of 92,502 follower‐weeks across 22 weeks. Furthermore, the campaign was implemented as planned in terms of posted material and continuation across 6 months. Breastfeed4Ghana was the first social media campaign designed specifically to protect, promote, and support breastfeeding in West Africa. Therefore, no information was available on the feasibility, acceptability, or impacts of such a campaign in Ghana, and we do not have comparative data or benchmarks to put into context the reach of this campaign. That said, Nestlé, a breast milk substitute company with a strong presence in Ghana, runs a Facebook page for its Ghanaian consumers that has been active since November 2015. Their Facebook page had 109,049 followers as of April 2019 (Nestle, 2015), putting into perspective the scale and potential for an infant and young child feeding social media campaign in Ghana, with greater time and resources. Many government, public health, and United Nation entities also have a social media presence that can be leveraged for health promotion purposes. Partnership with a reputable organization willing to house such a campaign could be an effective model to reduce the cost and time needed to acquire a campaign audience and may lead to a more sustainable campaign compared with the model conducted in the current study (Gough et al., 2017).

Paid advertising had a substantial impact on follower acquisition for Breasfeed4Ghana, as observed on both Facebook and Twitter. Each platform has advanced targeting mechanisms that can be utilized to effectively advertise to specific populations. The demographics of the Facebook followers are evidence that a combination of the targeted campaign advertisements and the campaign material and branding designed for 18‐ to 49‐year‐old Ghanaian adults was effective in acquiring the desired follower demographic for this campaign. It should be noted that the efficiency of the advertisements differs by platform and target population. For this campaign, the target population on Twitter was smaller compared with Facebook (i.e., less Ghanaian adults on Twitter than Facebook), and we found that paid advertisements were not as efficient at reaching the target population as seen on Facebook. It is also noteworthy that Twitter and Facebook have different algorithms for the content that appears on a user's feed and is dependent on how users engage with the different social media platforms (e.g., users who share specific content or like certain pages may receive more targeted advertisements as more user data are available on their interests; Facebook, 2019). Given the generally understood cost‐effective nature of online campaign delivery and the interest in using social media for health promotion (Yang, 2017, Allom et al., 2018), it is crucial that more data be generated on the actual cost and cost‐effectiveness of such campaigns (Korda & Itani, 2013, Johns, Lewis, & Langley, 2017).

Campaign acceptability was high based on the cross‐sectional survey, with over 90% of those who reported exposure to the campaign expressing positive descriptors of the campaign, such as informative, practical or useful, educational, or interesting. Similarly, campaign engagement was modest, as just under half (44%) of those who reported exposure to the campaign also reported sharing the campaign with others. Conversely, understanding of the campaign was low, with 61.0% of participants who reported exposure to the campaign also reporting not knowing or not remembering what the campaign was about. The mixed understanding of the purpose of the campaign, in conjunction with the 57% of those exposed to the campaign stating that the frequency of campaign material was not enough, suggests that campaign posts and messages were not regularly and widely delivered to the target audience. Alternatively, the high prevalence of nonparents in the survey sample may indicate that the breastfeeding campaign was not highly relevant to all who were exposed to it, which may explain why many could not remember what the campaign was about.

Given the low understanding of the campaign and presumed low frequency of exposure of the target audience to campaign messages, it is not surprising that no relationship was detected between campaign exposure and breastfeeding knowledge among cross‐sectional survey participants. From the in‐depth assessment of campaign engagement, acceptability, and understanding among those exposed to the campaign, it is clear that “exposure,” as defined in the current study, encompasses a heterogeneous exposure experience that could benefit from disaggregation. Although a sensitivity analysis to examine the relation of campaign understanding or engagement and breastfeeding knowledge would be informative, the sample size was not sufficient to perform such exploratory analyses. Further studies would benefit from more robust sample sizes that would allow analyses to better determine the relationship between campaign exposure and change in knowledge and the paths through which this relationship functions. A systematic review on the use of social media to deliver health promotion (yet to be published) noted the small sample sizes and short duration of several studies included (Johns et al., 2017).

Campaign understanding, exposure, and engagement may be increased with a higher frequency of posting of campaign materials and more precise targeting. However, boosting posts and generating paid advertisements would likely be more effective in reaching the target audience consistently. The campaign may also require more engaging materials, such as posts that prompt users to answer questions, or more videos. Alternatively, a behaviour change campaign to reduce skin cancer in Ireland that tested different message frames (i.e., humour, shock, informative, personal stories, and opportunistic) found that informative messages received the greatest number of shares, and humorous messages resulted in the greatest engagement (Gough et al., 2017).

With limited evidence from the health sector, we can learn from corporate marketing techniques and findings. In a 2015 review of creative strategies in social media marketing, Ashley and Tuten emphasize the need for branded content to be fresh and disseminated frequently, and the effective application of consumer incentives (Ashley & Tuten, 2015). For instance, Procter and Gamble's “Thank You Mom” campaign solicited user‐generated material by asking social media followers to share their stories, with the top stories “winning” the opportunity to have their story broadcasted (Ashley & Tuten, 2015). At the core of effective marketing is an understanding of consumer needs, motivations, and goals, for which Schmitt has proposed a conceptual model (Schmitt, 2012). Campaigns that target the consumer are likely to have greater success in achieving their desired impacts.

Based on the demographics of our Facebook page followers and those who took the online survey, there is a clear opportunity to reach a younger, perhaps preparenthood, population including both women and men. As noted by breastfeeding mothers in Brown's study, educating fathers, family society, and the next generation is key in breastfeeding support efforts (Brown, 2016). Thus, strategic market segmentation with tailored content for groups such as adolescent girls and boys, tailored to their specific needs, motivations, and goals, could offer an innovative approach to protecting, promoting, and supporting breastfeeding in the next generation.

There are no comparable breastfeeding social media campaigns that have published equivalent results to Breastfeed4Ghana. This was a limitation in the design of this study, as we did not have evidence from which we could generate reasonable expectations of feasibility or performance. This also limited our ability to generate an appropriate sample size calculation for the survey. For this survey, we did not have a priori knowledge on the feasibility of conducting an online, self‐administered survey in Ghana and recruiting for the survey on social media. Although online participant recruitment and survey administration for research is widespread in contexts such as the United States, no evidence in a comparable context existed. Due to this limitation, a conservative sample size was proposed based on feasibility.

Given the campaign exposure (0.32) and proportion of high breastfeeding knowledge among those not exposed to the campaign (44%), the sample size would have needed to double (*n* = 892) to detect a difference of 10% at an *α* = .05 and *β* = .2; of course, utilizing a highly sensitive, and possibly continuous, indictor for breastfeeding knowledge and narrowing the target population would drive the sample size down. Therefore, additional studies designed to rigorously examine the impacts of such campaigns are needed. The demographic of the sample should also be noted. The participants were a population of young, highly educated, and heavy social media users, which is not representative of Ghanaian adults of child‐bearing age, though possibly representative of social media users. Thus, the potentially biased sampling frame due to the internet‐based survey approach must be considered when interpreting the survey results (Wilson & Laskey, 2003); and future campaign should consider the “digital divide” and corresponding health disparities in planning the campaign and evaluation (Chou, Prestin, Lyons, & Wen, 2013). Furthermore, it is possible that as social media utilization continues to rise across Ghana, social media content will influence caregiver decisions and behaviours.

Despite these limitations, this study provides important data for future health‐focused social media campaigns in similar contexts. This study reveals that the first social media campaign in Ghana that aims to protect, promote, and support breastfeeding was highly feasible and acceptable. Such campaigns can provide a reliable and accurate source of information for the public in a sea of unreliable web‐based information (Chou et al., 2013). Like Breastfeed4Ghana, such campaigns must generate evidence‐informed content, and future campaigns should consider strategic investments in campaign promotion. It is also important to consider sustainability of a campaign, given evidence that online conversations around health promotion are *not* self‐sustaining (Syred, Naidoo, Woodhall, & Baraitser, 2014). Social media is a platform for health promotion and interventions that can be utilized at a relatively low cost to achieve high coverage, representing a unique opportunity for health behaviour intervention (Yang, 2017). Along with the opportunity for impacting health behaviour through social media, there is a great need to fill the gaps in the literature regarding best practices and impacts of such interventions in order for the health sector to successfully uptake and utilize social media most effectively for health promotion and education.

## CONFLICTS OF INTEREST

The authors declare that they have no conflicts of interest.

## CONTRIBUTIONS

KLH led the design of the research study with substantial contributions from RPE, RA, and GC. The research was led by KLH, RA, and RPE, with substantial contributions from GC, OL, and MY. OL and GC collected and managed the data, and KLH cleaned and analysed the data. KLH wrote the first manuscript draft manuscript; all authors reviewed and contributed to subsequent versions, read, and approved the paper submitted.
